# Efficacy of desmopressin in reducing post-procedural bleeding among chronic kidney disease patients: a systematic review and meta-analysis

**DOI:** 10.3389/fmed.2026.1857125

**Published:** 2026-08-26

**Authors:** Chananchida Sirilertmekasakul, Porpim Jindasakchai, Sudapree Sorasuchart, Tanat Lertussavavivat, Suramath Isaranuwatchai

**Affiliations:** 1 Faculty of Medicine, King Mongkut's Institute of Technology Ladkrabang, Bangkok, Thailand; 2 Department of Anesthesiology, King Chulalongkorn Memorial Hospital, The Thai Red Cross Society, Bangkok, Thailand; 3 Department of Anesthesiology, Faculty of Medicine, Chulalongkorn University, Bangkok, Thailand; 4 Department of Pharmacology, Faculty of Medicine, Chulalongkorn University, Bangkok, Thailand

**Keywords:** bleeding, chronic kidney disease, DDAVP, desmopressin, perioperative, procedure

## Abstract

**Introduction:**

Chronic kidney disease (CKD) is associated with uremic-related platelet dysfunction and a greater risk of perioperative bleeding. Desmopressin (DDAVP) is believed to improve platelet function and is used prophylactically to prevent post-operative bleeding, but with uncertain efficacy. In this research, we evaluated the efficacy of peri-procedural desmopressin administration in reducing bleeding complications in CKD patients.

**Methods:**

We searched PubMed, Scopus, and Embase up to August 2025 for randomized controlled trials (RCTs) and observational studies involving adult CKD patients undergoing non-surgical procedures who received peri-procedural desmopressin compared to a control group. Outcomes included overall bleeding events, hematoma, hematuria, hematocrit decline, transfusions, and interventions for bleeding control. Safety profile of hyponatremia was also assessed.

**Results:**

Of 1,744 records, a total of 7,944 participants from 21 studies were included. The meta-analysis showed that desmopressin did not significantly reduce overall bleeding events (OR 0.87, 95% CI 0.57–1.33). Similarly, there was no significant effect on the incidence of hematoma (OR 1.35, 95% CI 0.40–4.53) or hematuria (OR 1.44, 95% CI 0.50–4.12). Subgroup analysis restricted to randomized controlled trials (RCTs) demonstrated a significant reduction in overall bleeding events with desmopressin (OR 0.50, 95% CI 0.29–0.87). Further subgroup analysis by route of administration revealed that the intranasal route was associated with a significant reduction in total bleeding events (OR 0.52, 95% CI 0.32–0.84).

**Conclusion:**

This study suggests that desmopressin does not significantly reduce post-procedural bleeding in CKD patients. However, subgroup analyses restricted to RCTs, and the intranasal administration route suggest a reduction in bleeding complications.

**Systematic review registration:**

PROSPERO ID: CRD420251118342, URL: https://www.crd.york.ac.uk/PROSPERO/view/CRD420251118342.

## Introduction

Chronic kidney disease (CKD) is defined as abnormalities in kidney structure or function, present for a minimum of 3 months, with implications for health (1). As CKD progresses, the renal function declines and more uremic toxins accumulate in the patient’s body, resulting in an increased risk of bleeding (2). Studies have shown that there are inhibitions of platelet aggregation, clot formation, reduction in von Willebrand factor (vWF) synthesis, and deterioration of other adhesion molecules associated with coagulation pathways (3). This resulted in poor platelet adhesion, leading to impaired clot formation, and an increased bleeding tendency (2). Therefore, when CKD patients undergo many surgical or non-surgical procedures, such as kidney biopsy, central venous catheter placement, arteriovenous (AV) graft implantation, and hemodialysis, the risk of post-procedural bleeding is elevated, especially compared with patients with normal renal function (4).

Desmopressin acetate, also known as 1-deamino-8-D-arginine vasopressin (DDAVP), a synthetic vasopressin analog, is commonly used in the management of bleeding disorders, such as von Willebrand disease (5). It acts by stimulating vasopressin-2 receptors on endothelial cells, leading to the release of vWF and factor VIII, which enhances platelet vessel wall interactions (6). With these hemostatic effects, desmopressin is commonly administered preoperatively to reduce bleeding complications in CKD patients undergoing invasive procedures (7–9). However, the evidence for desmopressin in reducing post-procedural bleeding is limited and inconclusive (7–9). A meta-analysis by Wang et al. (10) suggested a potential benefit, but relied on studies with limited sample sizes, heterogeneous patient populations, and the absence of CKD-specific subgroup analysis, restricting the generalizability of its findings to the CKD population (1). While there are other proposed strategies to reduce peri-procedural bleeding risk in CKD patients such as cryoprecipitate or tranexamic acid, they are beyond scope of this meta-analysis (11). Thus, we conducted this study to evaluate the efficacy of desmopressin in reducing bleeding complications in CKD patients undergoing non-surgical procedures.

## Methods

### Data sources and searches

This study followed the Preferred Reporting Items for Systematic Reviews and Meta-Analyses (PRISMA) guidelines (12). The databases used in this study were PubMed, Scopus, and Embase. The search for the following databases was up to August 2025. The search strategies used include free-text terms for desmopressin, combined with free-text terms for different types of non-surgical procedures. Boolean operators, AND and OR, field tags, [MeSH] and [tiab], and wildcard (*) were used to broaden our search strategies. The search strings used in PUBMED, SCOPUS, and EMBASE databases are provided in Supplementary Table 1.

The protocol was registered in the International Prospective Register of Systematic Reviews (PROSPERO); ID CRD420251118342. Our study adhered to the principles of the Declaration of Helsinki and followed Good Clinical Practice guidelines.

### Eligibility criteria

The inclusion criteria for the selected studies were as follows: (1) available in English language, (2) included adults aged 18 years or older, (3) involved patients with CKD defined according to Kidney Disease: Improving Global Outcomes (KDIGO) criteria, (4) involved non-surgical procedures such as kidney biopsy, central venous catheter insertion, angiography, angioplasty, arteriovenous fistula/graft cannulation, or endoscopy, (5) included administration of perioperative desmopressin via any route such as intravenous, intranasal, or subcutaneous, (6) were clinical trials, cohort studies, case–control studies, or cross-sectional studies, and (7) reported outcomes on periprocedural bleeding complications—either as a composite or as individual components including hemoglobin drop, hematoma, gross hematuria, need for interventions to stop bleeding, or red blood cell transfusion—and/or safety outcomes including hyponatremia (1). Exclusion criteria included reviews, meta-analyses, letters, case reports, *in vitro* studies, and animal studies.

### Study selection and data extraction

Two authors (C.S. and P.J.) independently screened the relevant titles and abstracts of all the papers from PUBMED, SCOPUS and EMBASE using the search strings. The accepted studies were retrieved for full-text review. The authors (C.S. and P.J.) independently read and screened the studies according to the inclusion and exclusion criteria stated earlier. Disagreements were resolved through discussion with a third person for a consensus.

Two authors (C.S. and P.J.) independently extracted data from the included studies. The extracted information included: study title, authors’ names, publication date, study country, study design, number of participants, baseline characteristics [age, sex, serum creatinine, estimated glomerular filtration rate (eGFR)], route, dose, and timing of desmopressin administration, pre-procedural drugs discontinuation, types and details of procedures performed, and outcomes reported, including overall bleeding events, individual bleeding events consisting of hematoma, hematuria, blood transfusions and additional interventions to control bleeding, and non-bleeding events, consisting of hyponatremia, acute kidney injury, septicemia, length of hospital stay, and mortality. The overall bleeding events refer to the composite of all the aforementioned bleeding events reported in each study.

### Risk of bias and certainty assessment

The Cochrane Risk of Bias 2.0 (RoB 2) tool was used to assess the risk of bias in randomized controlled trials (RCTs) (13), while the Version 2 of Risk of Bias in Non-randomized Studies- of Interventions (ROBINS-I V2) tool was used for non-randomized studies (14). Two authors (C.S. and P.J.) independently assessed the risk of bias across the relevant domains for each included study.

The RoB 2 tool evaluates five domains: the randomization process, deviations from intended interventions, missing outcome data, outcome measurement, and selection of the reported result. The overall judgment is categorized as “low risk,” “some concerns,” or “high risk” of bias (13). The ROBINS-I V2 tool includes seven domains: confounding, selection of participants, classification of interventions, deviations from intended interventions, missing data, outcome measurement, and selection of the reported result. The overall risk of bias is rated as “low,” “moderate,” “serious,” or “critical” (14). Discrepancies between reviewers were resolved through discussion with a third person. The Grading of Recommendations Assessment, Development, and Evaluation (GRADE) approach was used to assess the overall certainty of evidence, which was rated as “high,” “moderate,” “low,” or “very low” (15).

### Synthesis methods

Dichotomous outcomes were reported as Odds Ratios (OR) with 95% Confidence Intervals (CI). A random-effects model was used for the meta-analysis, using inverse variance weighting to account for variability across studies. Between-study variance (*τ*^2^) was estimated using the restricted maximum-likelihood (REML) method, and 95% CI for τ^2^ and τ were derived using the Q-profile method. Statistical heterogeneity was assessed with Cochran’s Q test (Chi^2^) and quantified using the I^2^ statistic, with I^2^ more than 75% considered considerable heterogeneity. Pooled effect estimates are reported with 95% CI and illustrated using forest plots. Subgroup analysis was conducted on RCTs. Separately, we assessed the robustness of the primary analysis through two sensitivity analyses: a leave-one-out analysis for influential studies and an exclusion of outliers identified via the find.outliers function in R. All analyses were performed using R version 4.4.2 (R Foundation for Statistical Computing, Vienna, Austria) in RStudio 2025.5.0.496. Meta-analyses were conducted using the meta package version 8.0.2.

## Results

### Study selection

A total of 1,744 studies were identified through PUBMED, SCOPUS and EMBASE databases. After removing duplicates, 1,457 studies were retrieved for titles and abstracts screening. 58 articles remained for full-text review. Following a full-text review, 21 studies were included in this study. The selection process is shown in the PRISMA Flow in Supplementary Figure 1.

### Study characteristics

The 21 included studies, comprising 16 retrospective and prospective cohorts (7, 8, 16–29), and five RCTs (9, 30–33), were conducted between 1985 and 2025 across North America, Europe, Asia, and the Middle East. Sample sizes ranged from 23 to 3,018 participants from each study, summing up a total of 7,944 participants in this study. Eighteen studies performed percutaneous kidney biopsy (PKB), while other studies performed vascular access procedures (21), flexible bronchoscopy with biopsy (24), and endoscopy (29). Thirteen studies involved patients with mean eGFR less than 60 mL/min/1.73m^2^, three studies with eGFR more than 60 mL/min/1.73m^2^, while others did not report eGFR. eGFR of the patients ranged from less than 10 mL/min/1.73m^2^ to approximately 94 mL/min/1.73m^2^, with mean values of approximately 30 and 40 mL/min/1.73m^2^ in the desmopressin and control groups, respectively.

The intervention of interest was periprocedural desmopressin. Eleven studies administered via intravenous infusion [0.2–0.3 mcg/kg in most studies (16, 17, 19–24), or a fixed 20 mcg dose (18)], subcutaneous injection (0.3 mcg/kg) (9, 25, 26), or intranasal spray (150–300 mcg) (7, 8, 30–33). All studies except Kim et al. (21) have comparator groups consisting of patients who did not receive desmopressin within the same period. The primary outcome was composite bleeding complications, defined as the occurrence of any of the following: hemoglobin decline, hematuria, perinephric hematoma, blood transfusion requirement, or the need for additional interventions to control bleeding. Secondary outcomes included each individual bleeding component. The primary safety outcome was hyponatremia. Characteristics of the studies, procedures, and intervention details are shown in Table 1 and Supplementary Table 2, respectively. Characteristic details of intervention and control groups in each study are provided in Supplementary Table 3.

**Table 1:** Characteristics of studies included in the systematic review and meta-analysis.

Author	Year	Study design	Country	Sample size	Type of procedures	Comparator	Related outcomes	Other outcomes
Athavale et al. (16)	2019	Cohort study	USA	269	PKB	Control	Composite bleeding events	Hypotension
Sosa Barrios et al. (17)	2021	Cohort study	Spain	482	PKB	Control	Composite bleeding events	N/A
Bifari et al. (18)	2025	Cohort study	Saudi Arabia	410	PKB	Control	Composite bleeding events	N/A
Chakrabarti et al. (30)	2025	Double-blinded RCT	India	152	PKB	Control	Composite bleeding events	Size of perinephric hematoma
Cheong et al. (19)	2022	Cohort study	South Korea	3,018	PKB	Control	Composite bleeding events	Post-biopsy BP
Ho et al. (20)	2020	Cohort study	Singapore	195	PKB	Control	Composite bleeding events	Hyponatremia, thrombosis
Jose et al. (7)	2022	Cohort study	India	432	PKB	Control	Composite bleeding events	Length of stay
Kim et al. (21)	2015	Cohort study	South Korea	23	C-line, PKB, angiography	None	Composite bleeding events	N/A
Leclerc et al. (22)	2020	Cohort study	Canada	418	PKB	Control	Composite bleeding events	Hypotension, AKI
Lim et al. (23)	2019	Cohort study	Singapore	436	PKB	Control	Composite bleeding events	Severe hyponatremia
Manno et al. (9)	2011	Double-blinded RCT	Italy	162	PKB	Control	Composite bleeding events	Length of hospital stay
McCartney et al. (29)	2016	Cohort study	USA	99	Endoscopy	Control	Blood transfusion	Length of stay
Mehta et al. (24)	2005	Cohort study	USA	25	Bronchoscopy with biopsy	Control	Post-bronchoscopic events	N/A
Peters et al. (25)	2018	Cohort study	Sweden	527	PKB	Control	Composite bleeding events	N/A
Peters et al. (26)	2020	Cohort study	Sweden	375	PKB	Control	Composite bleeding events	Acute renal obstruction, sepsis
Prasad et al. (31)	2025	Double-blinded RCT	India	203	PKB	Control	Composite bleeding events	Hypotension
Rao and Chandra (8)	2020	Cohort study	India	194	PKB	Control	Composite bleeding events	N/A
Sateriale et al. (28)	1991	Cohort study	USA	241	PKB	Control	Composite bleeding events	N/A
Sattari et al. (32)	2022	Double-blinded RCT	Iran	120	PKB	Control	Composite bleeding events	Hyponatremia, hypotension
Sethi et al. (33)	2024	Double-blinded RCT	India	80	PKB	Control	Composite bleeding events	N/A
Tan et al. (27)	2022	Cohort study	Singapore	83	PKB	Control	Composite bleeding events	ESRD, death

RCT, Randomized Controlled Trial; PKB, Percutaneous Kidney Biopsy; AKI, Acute Kidney Injury; N/A, Not Available.

### Risk of bias in studies

The risk-of-bias evaluation using ROBINS-I V2 and Cochrane RoB 2 are shown in Supplementary Table 4. For non-randomized controlled studies, the overall risks of bias were moderate in one study (19), serious in 17 studies, and critical in three studies (21, 28, 29). The overall risk of bias for the randomized controlled studies was low in four studies and of some concern in one study (33). Publication bias was assessed using Funnel plot visual analysis. The figures can be found in Supplementary Figure 2.

### Results of syntheses

#### Primary outcome: composite bleeding events

**Figure 1:**
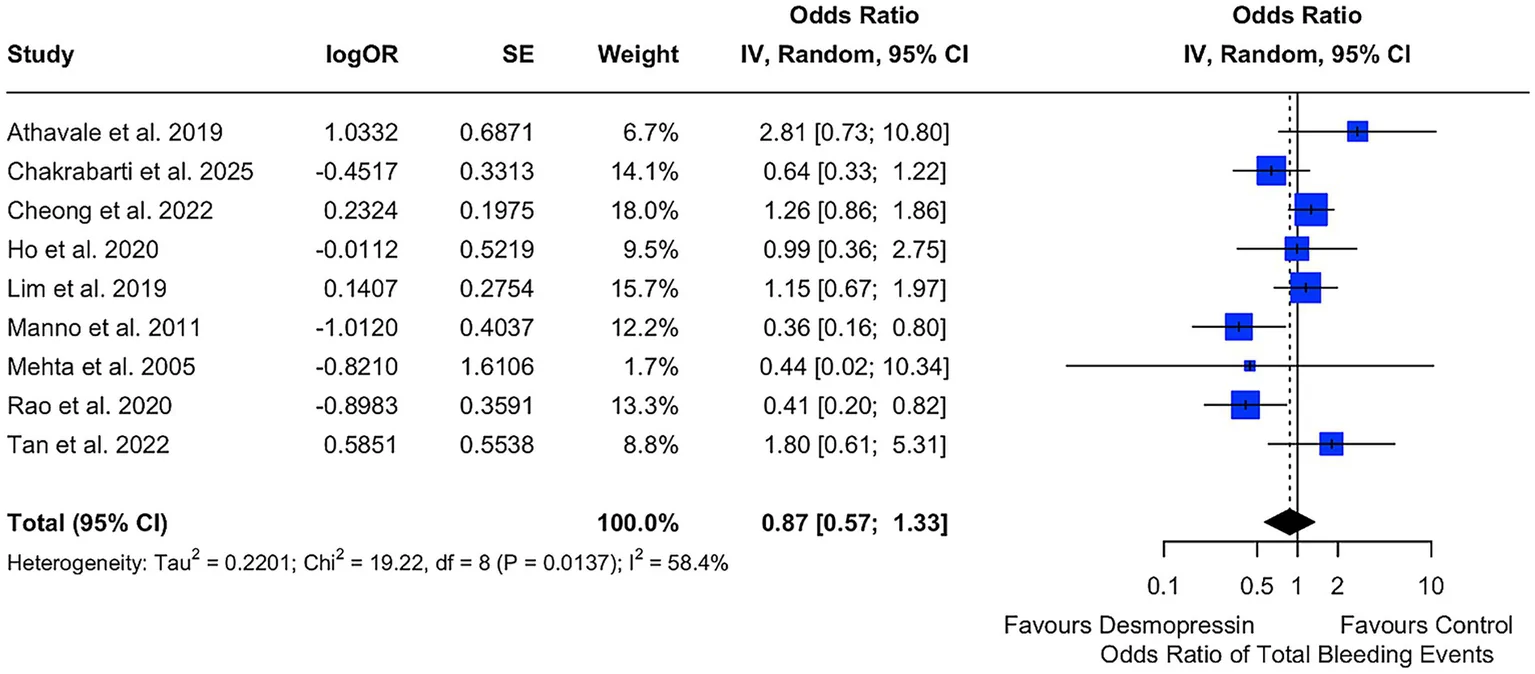
Forest plot of total bleeding outcomes. Forest plot of total bleeding outcomes comparing desmopressin with control showed that desmopressin was not associated with a significant reduction in bleeding events—pooled OR 0.87 (95% CI 0.57–1.33), *I^2^* = 58.4%. Each study is represented by a square, having the size proportional to its weight in the meta-analysis, and horizontal lines represent the 95% CI. The pooled OR is calculated using a random-effects model and is displayed with a diamond shape at the bottom of the plot. An OR <1 favors desmopressin, while an OR >1 favors control. logOR, natural logarithm of the Odds Ratio; SE, Standard Error; IV, Inverse Variance; CI, Confidence Interval; Tau^2^(τ^2^), Between-study variance; Chi^2^, Cochran’s Q test; df, degrees of freedom; I^2^, I^2^ statistic.

Thirteen studies reported data regarding composite bleeding outcomes, but four studies included patients with acute kidney injury, leaving only nine studies eligible for meta-analysis of perioperative bleeding outcomes in CKD patients. The analysis of the total bleeding outcome did not detect any outlier studies, showing the pooled estimate was consistent. The pooled random-effects analysis showed no significant difference in bleeding events between groups (OR 0.87, 95% CI 0.57–1.33), with moderate heterogeneity (I^2^ = 58.4%, *p* = 0.0137). The forest plot and GRADE evaluation are shown in Figure 1 and Supplementary Table 5, respectively. The Leave-One-Out analysis demonstrated that the pooled estimate was not disproportionately influenced by any single study. Furthermore, no statistical outliers were detected. Taken together, these findings support the robustness of our result. The figures can be found in Supplementary Figure 3.

### Subgroup analyses

#### Study design

**Figure 2:**
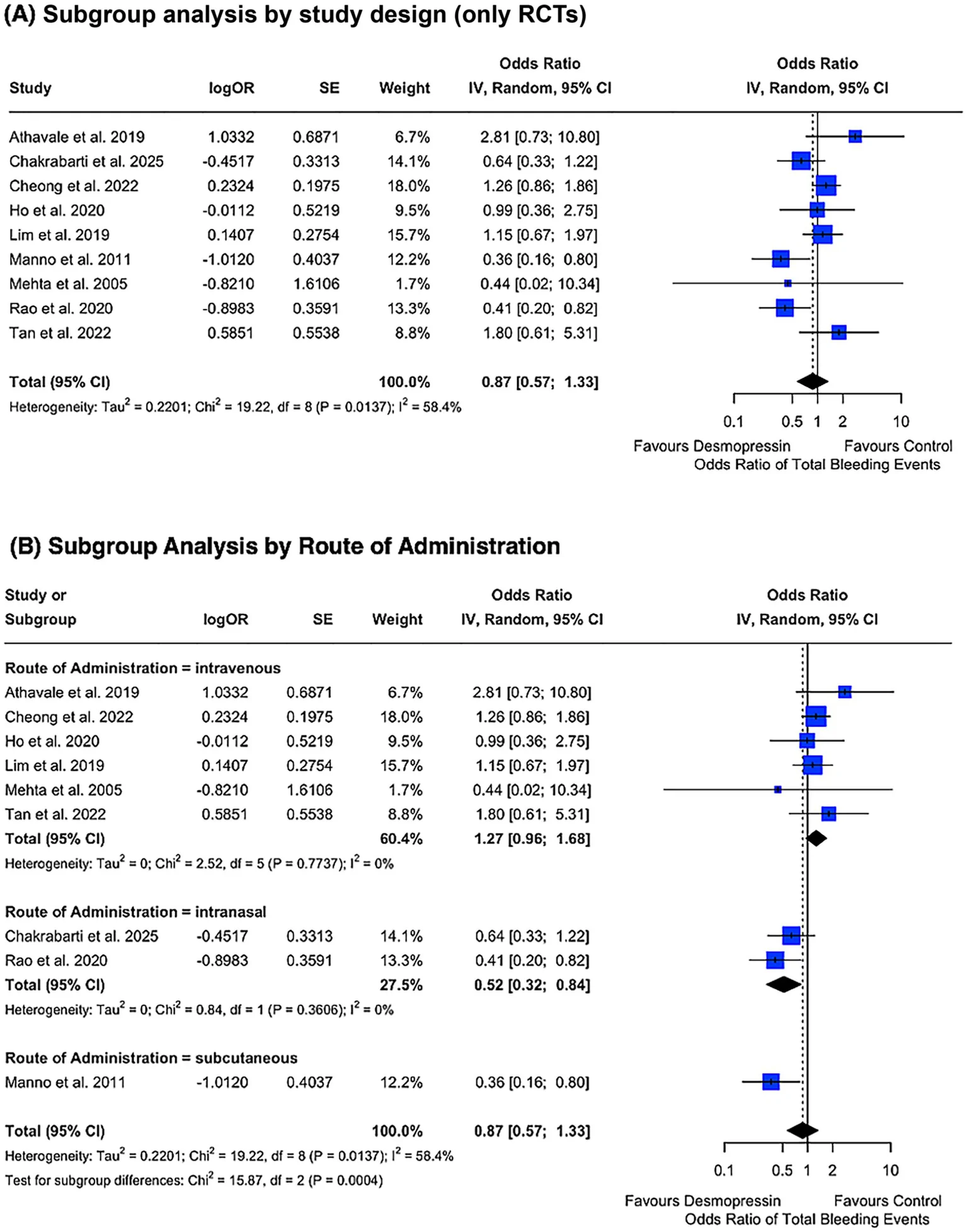
Subgroup analysis. **(A)** Subgroup analysis by study design (only RCTs). Subgroup analysis of total bleeding events with desmopressin versus control in RCTs showed a significant reduction in bleeding with desmopressin—pooled OR 0.50 (95% CI 0.29–0.87), I^2^ = 13.2%. Each study is represented by a square, having the size proportional to its weight in the meta-analysis, and horizontal lines represent the 95% CI. The pooled OR is calculated using a random-effects model and is displayed with a diamond shape at the bottom of the plot. An OR <1 favors desmopressin, while an OR >1 favors control. IV, Inverse Variance; CI, Confidence Interval; Tau^2^(τ^2^), Between-study variance; Chi^2^, Cochran’s Q test; df, degrees of freedom; I^2^, I^2^ statistic. **(B)** Subgroup analysis by route of administration. Forest plot of subgroup meta-analysis examining the association between desmopressin administration and overall bleeding events, stratified by route of administration. Odds ratios (ORs) with 95% confidence intervals (CIs) are shown for individual studies and pooled estimates using a random-effects model. Subgroup analyses were performed for intravenous, intranasal, and subcutaneous routes. Intranasal desmopressin was associated with a significant reduction in overall bleeding events, whereas no significant effect was observed with intravenous or subcutaneous administration. The vertical line represents no effect (OR = 1). Each study is represented by a square, having the size proportional to its weight in the meta-analysis, and horizontal lines represent the 95% CI. The pooled OR is calculated using a random-effects model and is displayed with a diamond shape at the bottom of the plot. An OR <1 favors desmopressin, while an OR >1 favors control. IV, Inverse Variance; CI, Confidence Interval; Tau^2^(τ^2^), Between-study variance; Chi^2^, Cochran’s Q test; df, degrees of freedom; I^2^, I^2^ statistic.

Of the seven observational studies, six were retrospective, and one was retrospective–prospective. The pooled analysis showed that these studies did not significantly reduce overall bleeding (OR 1.07 95% CI 0.68–1.67). In contrast, pooled analysis of two RCTs, including 154 patients with CKD in the desmopressin group and 160 controls, demonstrated a significant reduction in the risk of bleeding with desmopressin compared with control (OR 0.50, 95% CI 0.29–0.87). Heterogeneities were moderate among observational studies (I^2^ = 46.3%, *p* = 0.0834) and low among RCTs (I^2^ = 13.2%, *p* = 0.283). The result is depicted in Figure 2A.

#### Route of administration

We also performed subgroup analysis according to route of administration. Six studies administered desmopressin intravenously, while two studies administered intranasally. The results have shown that the intravenous route does not have a significant effect on the incidence of total bleeding events (OR 1.27, 95% CI 0.96–1.68), while the intranasal route can significantly decrease risk of total bleeding events (OR 0.52, 95% CI 0.32–0.84). Heterogeneities were low for both intravenous (I^2^ = 0%, *p* = 0.774) and intranasal routes (I^2^ = 0%, *p* = 0.361). The result is depicted in Figure 2B. Three studies administered desmopressin subcutaneously (0.3 mcg/kg); however, only Manno et al. (9) reported composite bleeding outcomes in CKD patients suitable for analysis, while the other two studies reported outcomes as composite major and minor biopsy complications without distinguishing bleeding from non-bleeding events. As a pooled subgroup estimate requires a minimum of two eligible studies, a subcutaneous route subgroup analysis could not be performed (9, 25, 26).

### Secondary outcomes

#### Hematoma

**Figure 3:**
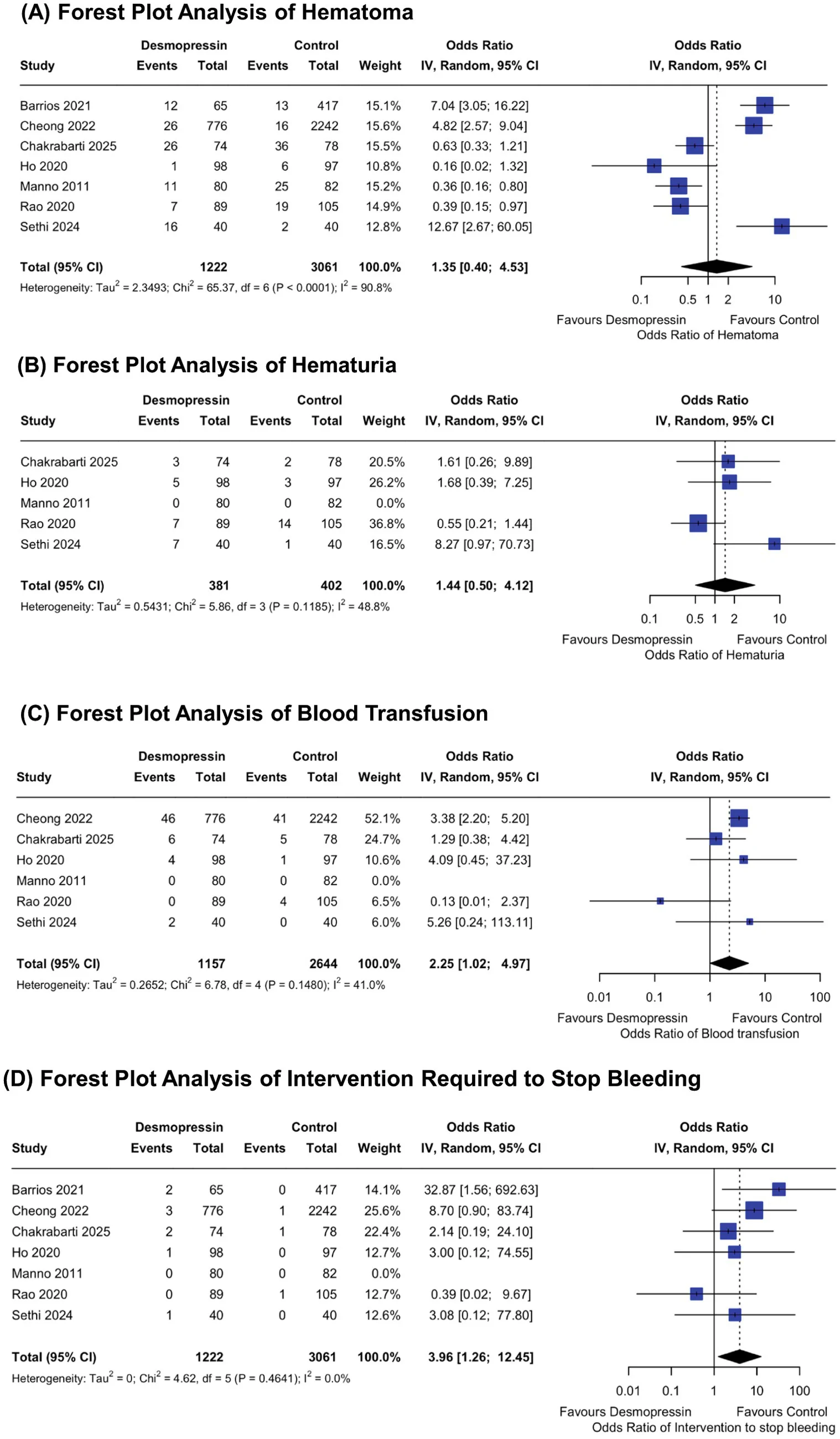
Forest plot analysis of secondary outcomes. Forest plots of secondary bleeding outcomes comparing desmopressin with control: **(A)** Hematoma—pooled OR 1.35 (95% CI, 0.40–4.53), *I^2^* = 90.8%; **(B)** Hematuria—pooled OR 1.44 (95% CI, 0.50–4.12), *I^2^* = 48.8%; **(C)** Blood transfusion—pooled OR 2.25 (95% CI, 1.02–4.97), *I^2^* = 41.0%; **(D)** Intervention required to stop bleeding—pooled OR 3.96 (95% CI, 1.26–12.45), *I^2^* = 0.0%. Each study is represented by a square, having the size proportional to its weight in the meta-analysis, and horizontal lines represent the 95% CI. The pooled OR is calculated using a random-effects model and is displayed with a diamond shape at the bottom of the plot. An OR <1 favors desmopressin, while an OR >1 favors control. IV, Inverse Variance; CI, Confidence Interval; Tau^2^(τ^2^), Between-study variance; Chi^2^, Cochran’s Q test; df, degrees of freedom; I^2^, I^2^ statistic.

We also performed meta-analyses of individual bleeding events. Seven studies performed post-procedural hematoma analysis in CKD patients. The pooled random-effects analysis revealed no significant difference in hematoma between the desmopressin and control groups (OR 1.35, 95% CI 0.40–4.53). Heterogeneity was high (I^2^ = 90.8%, *p* < 0.0001). The data is depicted in Figure 3. Subgroup and sensitivity analyses were performed to explore the sources of heterogeneity. Subgroup analysis by study design demonstrated similar insignificant effects in observational studies (OR 1.39, 95% CI 0.24–8.08) and RCTs (OR 1.29, 95% CI 0.17–10.06). Heterogeneity was high across studies (I^2^ = 90.8%, *p* < 0.0001). We further examined whether the route of desmopressin delivery influences the post-biopsy hematoma risk. In studies using intravenous desmopressin, the pooled analysis showed no significant difference compared with control (OR 2.15, 95% CI 0.25–18.25), with high heterogeneity (I^2^ = 81.2%, *p* = 0.0049). Similarly, intranasal administration was not associated with reducing hematoma risk (OR 1.32, 95% CI 0.17–10.09). The figures can be found in Supplementary Figure 4. In sensitivity analyses, it showed that heterogeneity increased further after excluding studies at high risk of bias (I^2^ = 92.4%, *p* < 0.0001). No influential outliers were identified using statistical outlier package detection in R. The figure can be found in Supplementary Figure 5.

#### Hematuria

Five studies analysed the post-procedural hematuria outcome in CKD patients. The pooled random-effects analysis showed no significant difference in hematuria between desmopressin and control groups (OR 1.44, 95% CI 0.50–4.12). Moderate heterogeneity was observed (I^2^ = 48.8%, *p* = 0.12). The result is shown in Figure 3.

#### Post-procedural blood transfusion

Six studies analysed post-procedural blood transfusion in CKD patients. The pooled analysis revealed that desmopressin significantly increased the odds of blood transfusion compared to the control group (OR 2.25, 95% CI 1.02–4.97). Moderate heterogeneity was observed (I^2^ = 41%, *p* = 0.148). The result is shown in Figure 3.

#### Bleeding-control intervention

Seven studies analysed intervention control bleeding. A pooled analysis demonstrated higher odds of intervention in the desmopressin group compared with the control group (OR 3.96, 95% CI 1.26–12.45). There was no evidence of heterogeneity among studies (I^2^ = 0%, *p* = 0.464). The result is shown in Figure 3.

#### Post-procedural drop of hemoglobin level

Three studies performed an analysis of the post-procedural drop of hemoglobin; however, none of these studies performed subgroup analysis in CKD patients. Therefore, a meta-analysis cannot be performed for this outcome.

#### Safety outcome: hyponatremia

Three studies evaluated the risk of hyponatremia. The pooled analysis revealed a significant association between desmopressin and an increased risk of hyponatremia compared with the control group (OR 2.61, 95% CI 1.36–5.01). Moderate heterogeneity was observed (I^2^ = 34.6%, *p* = 0.216). As we look thoroughly in each study, both Ho et al. (20) and Lim et al. (23) have an average pre-procedural serum sodium level of 137–138 mmol/L in both groups. Study from Ho et al. (20) measured serum sodium within three post-procedural days, but did not mention the definite onset of each case. Also, the study reported that 43 patients in the desmopressin group and 27 in the control group developed post-procedural hyponatremia (serum sodium <135 mmol/L). Furthermore, seven patients in the desmopressin group experienced severe hyponatremia (serum sodium <125 mmol/L), whereas none in the control group did. Among those with severe hyponatremia in the desmopressin group, three developed symptoms: two experienced mild symptoms such as headache and nausea, and one experienced a seizure (20). For Lim et al. (23) only severe hyponatremia, serum sodium ≤125 mmol/L was monitored within seven post-procedural days. The study showed that the sodium level was lowest at postoperative days two to five. There were 24 patients in the desmopressin group, and six controls who developed severe hyponatremia. Both studies further performed multivariate logistic regression to evaluate factors affecting serum sodium levels. They consistently showed that desmopressin is significantly associated with post-procedural hyponatremia, and that each 1 mmol/L increase in pre-procedural serum sodium reduced the hyponatremia incidence (23). In addition, Ho et al. (20) reported that patients who developed post-procedural hyponatremia had lower baseline serum sodium levels (137 mmol/L vs. 139 mmol/L), with the difference being statistically significant. They further demonstrated that each 100 mL increase in fluid intake was independently associated with a higher incidence of hyponatremia (20). Manno et al. (9) showed that none of the patients developed hyponatremia, thus their result was not included in the meta-analysis. The result is illustrated in Supplementary Figure 6.

## Discussion

Our study is one of the first systematic reviews and meta-analyses to evaluate the efficacy of perioperative desmopressin in reducing post-procedural bleeding in CKD patients. Across 21 studies including 7,944 patients, desmopressin was not shown to reduce overall post-procedural bleeding in CKD patients. However, subgroup analyses restricted to RCTs and the intranasal route of administration suggest a reduction in bleeding complications. Importantly, this study also identified a significant association between desmopressin use in CKD patients and the development of hyponatremia.

Prior systematic reviews and meta-analyses have shown a potential role for desmopressin in reducing bleeding in high-risk procedures. For example, Lim et al. (23) and Wang et al. (10) demonstrated reductions in bleeding events and blood loss, though without consistent reductions in reoperation rates. The present study advances beyond Wang et al. in several key respects. First, we exclusively enrolled CKD patients defined by KDIGO criteria directly addressing the gap Wang et al. (10) explicitly identified (1). According to the KDIGO criteria, CKD is stratified using a combined GFR and albuminuria staging system. GFR categories include G1 (≥90 mL/min/1.73 m^2^, normal or high), G2 (60–89, mildly decreased), G3a (45–59, mildly to moderately decreased), G3b (30–44, moderately to severely decreased), G4 (15–29, severely decreased), and G5 (<15 mL/min/1.73 m^2^, kidney failure); while albuminuria categories are classified as A1 (<30 mg/g), A2 (30–300 mg/g), and A3 (>300 mg/g) (1). Second, we included both RCTs and observational studies, with subgroup analysis by study design. Third, we performed subgroup analysis by route of administration, identifying intranasal desmopressin as the only route associated with significant bleeding reduction. Finally, we demonstrated a statistically significant hyponatremia signal in CKD patients, for which Wang et al. (10) found evidence in a general surgical population. Individual RCTs also reported reduced hematoma formation with intranasal desmopressin across a range of baseline kidney function (31, 32). In contrast, our pooled analysis did not demonstrate a significant overall reduction in bleeding among CKD patients, consistent with findings from a recent meta-analysis by Sartori Pacini et al. (34) which also reported inconsistent bleeding reduction. Moreover, desmopressin use was associated with a significantly increased risk of requiring blood transfusions and additional interventions to control bleeding in CKD patients. Several factors may explain this disagreement; first, the heterogeneity among included studies, including differences in study design and variations in baseline bleeding risk, may have influenced the pooled outcomes. The inclusion of retrospective studies, prospective cohorts, and RCTs introduces differences in confounding control, patient selection, and treatment effect estimation. In addition, inconsistent exclusion of high-risk patients across studies, such as chronic liver disease, coagulopathy, or concurrent antiplatelets or anticoagulants use, likely resulted in heterogeneous baseline bleeding profiles. Second, definitions and thresholds for transfusion and bleeding interventions were not standardized across studies, with some relying on clinical judgment, introducing potential bias. Moreover, patients receiving desmopressin, particularly in cohort studies, often had lower baseline hemoglobin levels and were therefore more likely to require transfusion. Importantly, desmopressin was frequently administered to patients with higher bleeding risk, such as those with thrombocytopenia, elevated urea, or impaired renal function, which may partly account for the increased need for additional interventions. Finally, the lower eGFR observed in desmopressin groups may have further contributed to bleeding risk, as uremic platelet dysfunction worsens with declining renal function (11, 35, 36). To address these concerns, we performed a subgroup analysis by study design. The result revealed a significant reduction in postoperative bleeding events with desmopressin use in studies restricted to RCTs, which included only studies with low risk of bias. This finding aligns with the hypothesis that study quality and appropriate patient selection are critical to observing therapeutic benefits. In clinical practice, this suggests that desmopressin may still be effective in selected CKD patients, particularly when used perioperatively in a controlled, evidence-based manner, and when bleeding risk is elevated due to uremic platelet dysfunction rather than surgical complexity alone.

Subgroup analyses by route of administration demonstrated a significant reduction in overall bleeding events with intranasal desmopressin. Six studies in this meta-analysis evaluated intranasal desmopressin; four studies employed a fixed dose ranging from 150 to 300 mcg, while two studies employed weight-based dosing of 3 mcg/kg (7, 8, 30–33). The administration routes and dosing regimens of all included studies are summarized in Supplementary Table 2. No consistent route-specific serious adverse events were reported across the included studies. A significant reduction in overall bleeding events with intranasal desmopressin suggests potentially greater hemostatic efficacy compared with intravenous administration, possibly reflecting pharmacokinetic differences. Prior studies have shown that peak activity occurs earlier with intravenous dosing (30–60 min) than with intranasal or subcutaneous administration (60–90 min), with the latter routes exhibiting a more prolonged effect (11). The more prolonged effect associated with intranasal and subcutaneous routes is further supported by studies demonstrating a reduction in hematoma size at 24 h post-biopsy (11). This sustained effect may be related to enhanced platelet adhesion mediated by collagen and thrombin activation following desmopressin administration. However, potential sources of bias should be considered. First, in the study by Rao and Chandra (8) which employed intranasal desmopressin, all patients receiving antiplatelet or anticoagulant therapy discontinued these medications at least 2 weeks prior to the procedure, potentially reducing baseline bleeding risk. Also, given the limited number of studies evaluating intranasal desmopressin, these findings should be interpreted with caution. Clinicians should therefore individualize the choice of administration route based on clinical context, anticipated benefits, and potential adverse effects.

Postoperative hematoma is a common complication, but findings across studies were inconsistent, with some reporting increased risk with desmopressin and others showing no effect or benefit, resulting in a high heterogeneity in the pooled analysis. This heterogeneity is likely attributable to several factors. This variability likely reflects inconsistent hematoma definitions, differences in postoperative imaging practices, and heterogeneous baseline bleeding risk due to varying eligibility criteria. Subgroup and sensitivity analyses did not resolve this heterogeneity, suggesting that methodological differences rather than a true treatment effect may account for the inconsistency.

The relationship between baseline kidney function and desmopressin efficacy deserves consideration. The two studies demonstrating statistically significant bleeding reduction were Manno et al. (9) (mean eGFR approximately 92 mL/min/1.73m^2^; OR 0.36, 95% CI 0.16–0.80) and Rao et al. (8) (mean eGFR approximately 36 mL/min/1.73m^2^; OR 0.41, 95% CI 0.20–0.82), representing markedly different levels of kidney function. In contrast, studies enrolling patients with more severely impaired kidney function (mean eGFR <30 mL/min/1.73m^2^) did not demonstrate significant benefit, with point estimates scattered on both sides of the null (20, 23, 27, 30). The results are displayed as forest plot in Figure 1. This does not support a simple linear relationship between eGFR and desmopressin efficacy. A plausible explanation is that in mild-to-moderate CKD, uremic platelet dysfunction remains partially reversible by desmopressin-mediated vWF release, whereas in severe CKD, the degree of uremic dysfunction may exceed corrective capacity of desmopressin (11, 35). However, a formal eGFR-based subgroup analysis was not feasible due to the limited number of studies reporting eGFR and heterogeneity in its measurement. This observation is therefore exploratory, and future studies should prospectively stratify patients by KDIGO CKD stage to evaluate whether kidney function modifies the hemostatic response to desmopressin.

Hyponatremia was the primary safety outcome assessed. Desmopressin use was associated with a significantly increased risk of hyponatremia, consistent with its V2 receptor–mediated antidiuretic effect (30). Hyponatremia is classified as mild (130–134 mmol/L), moderate (125–129 mmol/L), or severe (<125 mmol/L), with treatment indicated for symptomatic patients or those with rapidly declining serum sodium regardless of the absolute level. Fluid restriction is the first-line treatment for mild-to-moderate desmopressin-induced hyponatremia, whereas 3% hypertonic saline is reserved for severe or symptomatic cases (37). According to Ho et al. (20) restricting fluid intake to less than one liter is associated with reduction the incidence of hyponatremia; therefore, a recommendation for fluid restriction of less than one liter over 24 h, after DDAVP administration for renal biopsy should be considered. They also demonstrated that the incidence of post-procedural hyponatremia was significantly higher in patients with a baseline sodium of 137 mmol/L compared with 139 mmol/L. Although no validated prediction model exists for peri-biopsy desmopressin-induced hyponatremia, baseline serum sodium <139 mmol/L and post-procedural fluid intake >1 L within 24 h have consistently been identified as the strongest risk factors and may aid clinical risk stratification (20, 23). Therefore, maintaining serum sodium before the administration of desmopressin at or above 139 mmol/L and limiting post-procedural fluid intake are advisable.

This study demonstrated both statistical and clinical significance of hyponatremia, with both studies reporting higher rates of severe and symptomatic cases in the desmopressin group. Accordingly, the risks and benefits of desmopressin must be carefully weighed. Although the association is consistent, the timing of hyponatremia onset remains uncertain. Ho et al. (20) reported that both mild and severe hyponatremia can develop as early as 3 days post-procedure, while Lim et al. (23) observed nadir sodium levels between postoperative days two and five. Together, these findings suggest that clinically relevant hyponatremia may occur within the first three postoperative days, with risk extending up to at least 5 to 7 days. Based on this, serum sodium monitoring for a minimum of 5 to 7 days after desmopressin administration is advisable. Routine reviews of concomitant medications that may potentiate hyponatremia are also essential. However, as this conclusion is based on only two studies, the findings should be interpreted cautiously. Further research is needed to define the optimal monitoring window and to establish evidence-based strategies for early detection and prevention in high-risk patients. Future studies should investigate whether the risk of hyponatremia varies by the route of desmopressin administration. Although intranasal desmopressin was associated with a significant reduction in bleeding events in our subgroup analysis, the included studies did not systematically evaluate route-specific adverse events. Therefore, future studies should evaluate the risk–benefit profile of intranasal desmopressin to determine whether its potential hemostatic benefits outweigh the risk of hyponatremia.

There are several strengths of our study. First, we performed a meta-analysis to evaluate the efficacy and safety of perioperative desmopressin exclusively in CKD population, which is a population that is prone to bleeding following procedural interventions. Second, this study is not only limited to kidney biopsy. It extends across a broader range of non-surgical procedures, for example, flexible bronchoscopy with biopsy, endoscopy, and central catheter insertion. Third, the subgroup analysis of RCT was done to provide a higher level of evidence and minimize the confounding inherent to observational studies. This study has several limitations. First, most of the studies included were observational, contributing to heterogeneity and increased risk of bias. However, we managed to perform subgroup analysis in RCTs with a lower risk of bias, giving an insight into the potential impact of study designs on the overall results of the study. Due to the discrepancy between overall and RCT analyses results, a well-designed RCT should be conducted to provide a definite answer. Also, most available evidence was limited to percutaneous kidney biopsy populations, and one study included a non-renal procedure (bronchoscopy), which restricts generalizability to other interventions. Further studies should focus more on procedures other than percutaneous kidney biopsy to broaden the applicability of desmopressin prophylaxis with other procedures. Besides that, long-term follow-up is necessary to study delayed outcomes along with a broader range of safety and efficacy outcomes, including optimal dose and route of administration, and subgroup analyses by CKD stage should be clarified to focus on specific risks and benefits.

## Conclusion

In this systematic review and meta-analysis, perioperative desmopressin did not significantly reduce overall post-procedural bleeding in CKD patients. Subgroup analyses of RCTs and intranasal route suggested a potential reduction in bleeding risk; however, these findings were not accompanied by consistent benefits on key clinical outcomes such as post-procedural hematoma, and the absolute risk reduction appears limited. Given the lack of consistent clinically significant benefit and the increased risk of hyponatremia, routine use of desmopressin is not recommended. Therefore, the decision for desmopressin administration should be individualized, carefully weighing the benefits against safety concerns in CKD patients. Further well-designed, adequately powered RCTs are needed to determine whether any subgroup-specific benefits translate into clinically relevant improvements in outcomes.

## Data Availability

The original contributions presented in the study are included in the article/Supplementary material, further inquiries can be directed to the corresponding author.
