# Supplementary material for: Efficacy of desmopressin in reducing post-procedural bleeding among chronic kidney disease patients: a systematic review and meta-analysis
Source: Front Med (Lausanne). 2026 Aug 26;13:1857125. doi: 10.3389/fmed.2026.1857125 (PMC13553829; doi:10.3389/fmed.2026.1857125)
Supplement: Supplementary file 1 [file Supplementary_file_1.docx]

**Supplementary File for
The Efficacy of Desmopressin in Reducing Post-Procedural Bleeding Among**

**Chronic Kidney Disease Patients: A Systematic Review and Meta-Analysis**

**Table of Contents**

[**Supplementary Table 1: Search Strings**	2](#_Toc219324514)

[**Supplementary Table 2: Procedures and DDAVP administration details**	3](#_Toc219324515)

[**Supplementary Table 3: Characteristic details of intervention and control groups**	5](#_Toc219324516)

[**Supplementary Table 4: Risk-of-Bias Evaluation**	6](#_Toc219324517)

[*(A) Risk of bias assessment in non-randomized studies using ROBINS-I V2 tool*	6](#_Toc219324518)

[*(B) Risk of bias assessment in randomized studies using Cochrane RoB 2 tool*	6](#_Toc219324519)

[**Supplementary Table 5: Assessment of Evidence Quality: the GRADE Framework**	7](#_Toc219324520)

[**Supplementary Figure 1:Preferred Reporting Items for Systematic Reviews and Meta-Analysis (PRISMA) Flow Diagram**	9](#_Toc219324521)

[**Supplementary Figure 2: Publication Bias Analysis Using Funnel Plot**	10](#_Toc219324522)

[**Supplementary Figure 3: Leave-One-Out Sensitivity Analysis**	11](#_Toc219324523)

[**Supplementary Figure 4: Subgroup Analyses of Hematoma Outcome**	12](#_Toc219324524)

[*(A) Subgroup Analysis of Hematoma Outcome by Route of Administration*	12](#_Toc219324525)

[*(B) Subgroup Analysis of Hematoma Outcome by Study Design*	12](#_Toc219324526)

[**Supplementary Figure 5: Sensitivity Analysis of Hematoma Outcomes**	13](#_Toc219324527)

[**Supplementary Figure 6: Forest Plot Analysis of Safety Outcome: Hyponatremia**	14](#_Toc219324528)

**Supplementary Table 1: Search Strings**

*PUBMED:*

Pubmed: (((Surgery OR Surgical) OR (((((((((((((((((((((((procedure[MeSH]) OR (procedures)) OR (biopsy[MeSH])) OR (biopsies)) OR (catheters[MeSH])) OR (procedure*)) OR (biops*)) OR (angiography[MeSH])) OR (angiography)) OR (angiograph*)) OR (fistula)) OR (fistula[MeSH])) OR (AVF OR Arteriovenous Fistula)) OR (AVG OR Arteriovenous Graft)) OR (intervention)) OR (intervention[MeSH])) OR (endoscopy)) OR (endoscopy[MeSH])) OR (catheter)) OR (catheter[MeSH])) OR (vascular access)) OR (central venous)))) AND ((ESRD ESKD) OR (((Transplant) OR (Dialysis)) OR (((Kidney OR Renal) OR (Chronic kidney)) OR (CKD))))) AND (((desmopressin[MeSH]) OR (DDAVP)) OR (Deamino Arginine Vasopressin))

*SCOPUS:*

( TITLE-ABS-KEY ( desmopressin or ddavp or "Deamino Arginine Vasopressin" ) and TITLE-ABS-KEY ( "chronic kidney disease" or ckd or "renal failure" or "kidney insufficiency" or "ESRD" or "dialysis" or "ESKD" or "transplant" ) and TITLE-ABS-KEY ( surgery or surgical or operation or operative or procedure* or biopsy or "kidney biopsy" or endoscop* or angiogra* or catheter* or "arteriovenous fistula" or "anteriovenous graft" or "vascular access" or "AVF" or "AVG") )

*EMBASE:*

1. (transplant or dialysis or kidney or renal or "chronic kidney" or CKD).mp. [mp=title, abstract, heading word, drug trade name, original title, device manufacturer, drug manufacturer, device trade name, keyword heading word, floating subheading word, candidate term word]
2. "surgical procedur*".mp.
3. exp biopsy/ or biops*.mp.
4. exp catheter/ or catheter.mp.
5. exp central venous catheter/ or central venous catheter.mp.
6. exp central venous catheterization/ or central venous catheterization.mp.
7. vascular access.mp. or exp vascular access/
8. exp endoscopy/ or endoscop*.mp.
9. exp angiography/ or angiograph*.mp.
10. exp angioplasty/ or angioplasty.mp.
11. exp arteriovenous fistula/ or arteriovenous fistula.mp. or "AV fistula".mp. or "AVF".mp. or "arterovenous graph".mp. or "AVG".mp. [mp=title, abstract, heading word, drug trade name, original title, device manufacturer, drug manufacturer, device trade name, keyword heading word, floating subheading word, candidate term word]
12. intervention.mp.
13. exp desmopressin/ or desmopressin.mp.
14. (DDAVP or "deamino arginine vasopressin").mp. [mp=title, abstract, heading word, drug trade name, original title, device manufacturer, drug manufacturer, device trade name, keyword heading word, floating subheading word, candidate term word]
15. 2 or 3 or 4 or 5 or 6 or 7 or 8 or 9 or 10 or 11 or 12
16. 13 or 14
17. 1 and 15 and 16

# **Supplementary Table 2: Procedures and DDAVP administration details**

| **Author** | **Pre-biopsy drug discontinuation** | **Type of procedures** | **Imaging guided** | **Gauge size** | **Administrator** | **DDAVP indications** | **DDAVP route** | **DDAVP dose** | **DDAVP pre-procedure** |
| --- | --- | --- | --- | --- | --- | --- | --- | --- | --- |
| Athavale | ASA:1week, clopidogrel:5 days, anticoagulants: expert opinion | PKB | US | 18G | Nephrologist | expert opinion | IV | 0.3 μg/kg | 30 min |
| Barrios | N/A | PKB | US | 14G/16G | Nephrologist | # | IV | 0.3 μg/kg | 60 min |
| Bifari | N/A | PKB | US | N/A | Nephrologist / IR | N/A | IV | 20 μg/ | 30-120 min |
| Chakrabarti | N/A | PKB | US | 16G | Nephrologist | N/A | IN | 3 μg/kg | 60 min |
| Cheong | ASA&clopidogrel: 7 days, cilostazol: 3 days, warfarin: until PT/INR normalized | PKB | US | 16G/ 18G | nephrologist, others | expert opinion | IV | 0.3 μg/kg | 30 min |
| Ho | N/A | PKB | US | 16G | Nephrologist / IR | * | IV | 0.3μg/kg | 60 min |
| Jose | antiplatelets & anticoagulants: 7 days | PKB | US | 18G | Nephrologist | N/A | IN | 160 μg | 15-60 min |
| Kim | N/A | C-line, PKB, angiography | - | - | N/A | N/A | IV | 0.3 μg/kg | 30 min |
| Leclerc | antiplatelets:7-10 days, anticoagulants: 5 days ^ | PKB | US & CT | 16G/18G | IR | expert opinion | IV | 0.3 μg/kg | N/A |
| Lim | N/A | PKB | US | 16G | Nephrologist / IR | * | IV | 0.1-0.4 μg/kg | 60 min |
| Manno | antiplatelets:7days, heparin: 1-2 days | PKB | US | 16G | Nephrologist | N/A | SC | 0.3 μg/kg | 60 min |
| McCartney | N/A | Endoscopy | - | - | N/A | N/A | N/A | N/A | N/A |
| Mehta | N/A | Bronchoscopy with biopsy | N/A | N/A | N/A | hemodialysis | IV | 0.3 μg/kg | 30 min |
| Peter | N/A | PKB | US | 18G | Nephrologist / IR | Cr > 150 μmol/L | SC | 0.3 μg/kg | N/A |
| Peter | N/A | PKB | US | 16G/18G | Nephrologist / IR | Cr > 150 μmol/L | SC | 0.3 μg/kg | 60 min |
| Prasad | N/A | PKB | US | 18G | Nephrologist | N/A | IN | 300 mg | 60 min |
| Rao | antiplatelets & anticoagulants: ≥ 2weeks | PKB | US | 16G/18G | Nephrologist | N/A | IN | 150 μg | 60 min |
| Sateriale | N/A | PKB | CT | 14G | IR | N/A | IV | N/A | N/A |
| Sattari | Antiplatelets: 7 days, heparin: 2 days | PKB | US | 18G | IR | N/A | IN | 3 μg/kg | 60 min |
| Sethi | N/A | PKB | US | 18G | Nephrologist | N/A | IN | 150 μg | 60 min |
| Tan | N/A | PKB | US | 16G | N/A | * | IV | N/A | 60 min |

Abbreviations: ASA: Aspirin; DDAVP: 1-deamino-8-D-arginine Vasopressin (Desmopressin); PKB: Percutaneous Kidney Biopsy; N/A: Not Available; -: Not Related; IR: Interventional Radiologist; IV: Intravenous; IN: Intranasal; SC: Subcutaneous;

#: Cr >176.84 mmol/L, eGFR<60mL/min/1.73m2, serum urea >24.9 mmol/L altered PFA-100 platelet <150,000/mm3;

*:serum urea >15 mmol/L, Cr >200 µmol/L , eGFR<30 ml/min/1.73 m2;

^: stopped medications as relevant guidelines [39]

# **Supplementary Table 3: Characteristic details of intervention and control groups**

| No. | Author | year | total number | total CKD | DDVAP group | | | | | | control group | | | | | |
| --- | --- | --- | --- | --- | --- | --- | --- | --- | --- | --- | --- | --- | --- | --- | --- | --- |
|  |  |  |  |  | total | CKD | Age (SD) | Female (%) | Cr (mg/dL) (SD) | eGFR (SD) | total | CKD | age (SD) | female (%) | Cr (mg/dL) (SD) | eGFR (SD) |
| 1 | Athavale | 2019 | 269 | 85 | 100 | 35 | 46.02 (14.44) | 55 (55.00%) | 3.34 (2.6) | 38.56 (38.81) | 169 | 50 | 48.22 (1.62) | 89 (52.66%) | 1.79 (1.38) | 64.93 (49.59) |
| 2 | Barrios | 2021 | 482 | N/A | 65 | N/A | 54.5 (14.1) | 22 (33.85%) | N/A | 27.25 (22.28) | 417 | N/A | 54 (15.9) | 23 (5.52%) | N/A | 50.13 (37.58) |
| 3 | Bifari | 2025 | 410 | 305 | 210 | 159 | 42.3 (14.7) | 84 (40.00%) | N/A | N/A | 204 | 146 | 42.9 (15.9) | 73 (35.78%) | N/A | N/A |
| 4 | Chakrabarti | 2025 | 152 | N/A | 74 | 74 | 39 (15) | 40 (54.05%) | 3.6 (1.5) | 23 (13) | 78 | 78 | 39 (16) | 34 (43.59%) | 3.7 (1.5) | 22 (12) |
| 5 | Cheong | 2022 | 3018 | N/A | 776 | 776 | 50.2 (16.8) | 344 (44.3) | 2.87 (2.68) | 45 (34.5) | 2242 | 2242 | 43.5 (16.1) | 1155 (51.50%) | 1.24 (1.25) | 73.6 (28.2) |
| 6 | Ho | 2020 | 195 | 195 | 98 | 98 | 50.6 (15.3) | 47 (47.96%) | 3.17 (1.48) | 19 (8.85) | 97 | 97 | 50.6 (13.8) | 39 (40.21%) | 2.15 (0.76) | 30.4 (14.7) |
| 7 | Jose | 2022 | 432 | 110 | 307 | N/A | 39.2 (15.4) | 114 (37.13%) | 6.07 (1.63) | 10.89 (3.71) | 125 | 125 | 38.9 (15.5) | 43 (34.40%) | 5.86 (1.63) | 11.62 (4.22) |
| 8 | Kim | 2015 | 23 | 23 | 23 | 23 | 60.2 (11.7) | 7 (30.43%) | 10.02 (4.52) | 6.1 (3.33) | 0 | 0 | 0 (0) | 0 (0.00%) | 0 (0) | 0 (0) |
| 9 | Leclerc | 2020 | 418 | 302 | 328 | 251 | 60 (19.3) | 144 (43.90%) | 2.2 (1.71) | 28 (26.7) | 85 | 51 | 64 (13.3) | 43 (50.59%) | 1.4 (0.89) | 45 (40) |
| 10 | Lim | 2019 | 436 | 436 | 226 | 226 | 51.5 (14.3) | 106 (46.90%) | 3.16 (1.6) | 16.9 (9.85) | 210 | 210 | 51.3 (12.4) | 82 (39.05%) | 2.12 (0.68) | 27.3 (13.7) |
| 11 | Manno | 2011 | 162 | N/A | 80 | 80 | 39.5 (14.2) | 35 (43.75%) | 1 (0.3) | 94.2 (22.8) | 82 | 82 | 41.7 (15) | 39 (47.56%) | 1 (0.2) | 89.4 (21.3) |
| 12 | Mehta | 2005 | 25 | 25 | 7 | 7 | N/A | N/A | 11.95 (3.375) | N/A | 18 | 18 | N/A (N/A) | N/A | 2.7 (0.9) | N/A |
| 13 | Peter | 2018 | 527 | 527 | 204 | N/A | 55.6 (16.6) | 53 (25.98%) | 3.82 (3.02) | 22.3 (11) | 372 | N/A | 58.5 (1.7) | 116 (31.18%) | 3.57 (2.31) | 22.4 (11) |
| 14 | Peter | 2020 | 375 | N/A | 39 | 39 | N/A | N/A | N/A | N/A | 243 | 243 | N/A | N/A | N/A | N/A |
| 15 | Prasad | 2025 | 203 | 15 | 101 | 9 | 38.53 (15.1) | 34 (33.66%) | 2.4 (1.93) | 30 (41.1) | 102 | 9 | 42.64 (15.13) | 42 (41.18%) | 2.47 (2.3) | 31 (38.4) |
| 16 | Rao | 2020 | 194 | N/A | 89 | N/A | 40.7 (16.8) | N/A | 2.33 (0.94) | 36.58 (15.33) | 105 | 105 | 36 (16.8) | N/A | 2.45 (0.89) | 34.92 (14.58) |
| 17 | Sattari | 2022 | 120 | 111 | 60 | 57 | 43 (16.9) | 29 (48.33%) | 1.57 (0.81) | 54.14 (20.52) | 60 | 54 | 46.85 (14.94) | 33 (55.00%) | 1.92 (1.2) | 49.41 (15.63) |
| 18 | Sethi | 2024 | 80 | 80 | 40 | 40 | 42.9 (14.35) | 14 (35.00%) | 4.19 (4.36) | 22.4 (12.37) | 40 | N/A | 45.25 (11) | 16 (40.00%) | 4.65 (2.83) | 19.23 (12.86) |
| 19 | Tan | 2022 | 83 | 83 | 48 | 48 | 66.6 (5.5) | 24 (50.00%) | 2.78 (1.43) | 17.5 (9.56) | 35 | 35 | 67 (5.7) | 26 (74.29%) | 2.24 (0.6) | 24.5 (7.78) |
| 20 | Sateriale | 1991 | 241 | N/A | 5 | N/A | N/A | N/A | N/A | N/A | 236 | N/A | N/A | N/A | N/A | N/A |
| 21 | McCartney | 2016 | 99 | N/A | 13 | N/A | N/A | N/A | N/A | N/A | 86 | N/A | N/A | N/A | N/A | N/A |
| **Mean eGFR (mL/min/1.73 m²)** | | | | | | | | | | **30.7 (DDAVP)** |  | | | | | **39.7 (Control)** |

Abbreviations: no information

# **Supplementary Table 4: Risk-of-Bias Evaluation**

## *(A) Risk of bias assessment in non-randomized studies using ROBINS-I V2 tool*

| Study | D1 | D2 | D3 | D4 | D5 | D6 | D7 | overall |
| --- | --- | --- | --- | --- | --- | --- | --- | --- |
| Athavale 2019 | 🔴 | 🟡 | 🟢 | 🟢 | 🟢 | 🟢 | 🟡 | 🔴 |
| Barrios 2021 | 🔴 | 🟡 | 🟢 | 🟢 | 🟢 | 🔴 | 🟡 | 🔴 |
| Bifari 2025 | 🔴 | 🟢 | 🟢 | 🟡 | 🟡 | 🟡 | 🟡 | 🔴 |
| Cheong 2022 | 🟡 | 🟡 | 🟢 | 🟢 | 🟡 | 🟢 | 🟢 | 🟡 |
| Ho 2020 | 🔴 | 🟢 | 🟢 | 🟡 | 🟢 | 🔴 | 🟡 | 🔴 |
| Jose 2022 | 🔴 | 🟡 | 🟢 | 🟢 | 🟢 | 🔴 | 🟡 | 🔴 |
| Kim 2015 | 🟤 | 🟢 | 🟢 | 🟢 | 🟢 | 🟡 | 🟡 | 🟤 |
| Leclerc 2020 | 🔴 | 🟢 | 🟢 | 🟢 | 🟡 | 🟡 | 🟡 | 🔴 |
| Lim 2019 | 🔴 | 🟡 | 🟢 | 🟡 | 🟡 | 🔴 | 🟡 | 🔴 |
| Mehta 2005 | 🔴 | 🔴 | 🟡 | 🟢 | 🟡 | 🟢 | 🟢 | 🔴 |
| Peter 2018 | 🔴 | 🟢 | 🟢 | 🟢 | 🟡 | 🟡 | 🟡 | 🔴 |
| Peter 2020 | 🔴 | 🟡 | 🟢 | 🟡 | 🟡 | 🟢 | 🟡 | 🔴 |
| Rao 2020 | 🔴 | 🟡 | 🟢 | 🟡 | 🟡 | 🟢 | 🔴 | 🔴 |
| Tan 2022 | 🔴 | 🟡 | 🟢 | 🟡 | 🟡 | 🔴 | 🟡 | 🔴 |
| Sateriale 1991 | 🟤 | 🔴 | 🟢 | 🟢 | 🟡 | 🟢 | 🟡 | 🟤 |
| McCartney 2016 | 🟤 | 🟡 | 🟢 | 🟢 | 🟡 | 🟢 | 🟡 | 🟤 |

Abbreviations: D = domain; D1 = confounding bias; D2 = selection into the study; D3 = classification of intervention; D4 = deviations from intended intervention; D5 = missing data; D6 = measurement of the outcome; D7 = selection of reported result; 🟢 = low risk; 🟡 = moderate risk; 🔴 = serious risk; 🟤 = critical

## *(B) Risk of bias assessment in randomized studies using Cochrane RoB 2 tool*

| Study | D1 | D2 | D3 | D4 | D5 | overall |
| --- | --- | --- | --- | --- | --- | --- |
| Chakrabarti2025 | 🟢 | 🟢 | 🟢 | 🟢 | 🟢 | 🟢 |
| Manno2011 | 🟢 | 🟢 | 🟢 | 🟢 | 🟢 | 🟢 |
| Prasad2025 | 🟢 | 🟢 | 🟢 | 🟢 | 🟢 | 🟢 |
| Sattari2022 | 🟢 | 🟢 | 🟢 | 🟢 | 🟢 | 🟢 |
| Sethi2024 | 🟡 | 🟢 | 🟢 | 🟢 | 🟢 | 🟡 |

Abbreviations: D = domain; D1 = randomization process; D2 = assignment to intervention; D3 = missing outcome data; D4 = measurement of the outcome; D5 = selection of the reported result; 🟢 = low risk; 🟡 = some concerns; 🔴 = high risk

| **Supplementary Table 5: Assessment of Evidence Quality: the GRADE FrameworkOutcomes** | **Participants (Studies)** | **Certainty Assessment** | | | | | **Hetero-geneity (%)** | **p value** | **Results (OR)** | **95% CI** | **Overall Certainty** |
| --- | --- | --- | --- | --- | --- | --- | --- | --- | --- | --- | --- |
|  |  | **Risk of Bias** | **Inconsistency** | **Indirectness** | **Imprecision** | **Publication bias** |  |  |  |  |  |
| Total bleeding events | 4534 (9) | serious | serious | not serious | serious | not serious | 58.4 | 0.0137 | 0.87 | 0.57–1.33 | ⊕◯◯◯  **VERY LOW** due to risk of bias, inconsistency and imprecision |
| Total bleeding (RCT) | 314 (2) | not serious | not serious | not serious | serious | not serious | 13.2 | 0.2832 | 0.5 | 0.29–0.87 | ⊕⊕⊕◯ **MODERATE** due to imprecision |
| Total bleeding (Observational) | 4220 (7) | serious | serious | not serious | serious | not serious | 46.3 | 0.0834 | 1.07 | 0.68-1.67 | ⊕◯◯◯  **VERY LOW** due to risk of bias, inconsistency and imprecision |
| Total Bleeding  (Route: IV) | 4026 (6) | serious | not serious | not serious | serious | not serious | 0 | 0.7737 | 1.27 | 0.96-1.68 | ⊕⊕◯◯  **LOW** due to risk of bias and imprecision |
| Total Bleeding  (Route: NS) | 346 (2) | serious | not serious | not serious | not serious | not serious | 0 | 0.3606 | 0.52 | 0.32-0.84 | ⊕⊕⊕◯ **MODERATE** due to risk of bias |
| Hematuria | 783 (5) | not serious | serious | not serious | serious | not serious | 48.8 | 0.1185 | 1.44 | 0.50-4.12 | ⊕⊕◯◯  **LOW** due to inconsistency and imprecision |
| Hyponatremia | 793 (3) | not serious | not serious | not serious | not serious | serious | 34.6 | 0.148 | 2.61 | 1.36–5.01 | ⊕⊕⊕◯ **MODERATE** due to publication bias |
| Intervention to stop bleeding | 4283 (7) | serious | not serious | not serious | serious | not serious | 0 | 0.4641 | 3.96 | 1.26–12.45 | ⊕⊕◯◯  **LOW** due to risk of bias and imprecision |
| Blood transfusion | 3801 (6) | serious | not serious | not serious | serious | not serious | 41 | 0.2182 | 2.25 | 1.02 – 4.97 | ⊕⊕◯◯  **LOW** due to risk of bias and imprecision |
| Hematoma | 4283 (7) | serious | very serious | not serious | serious | not serious | 90.8 | <0.0001 | 1.35 | 0.40-4.53 | ⊕◯◯◯  **VERY LOW** due to risk of bias, inconsistency and imprecision |

Abbreviations: CI: Confidence Interval; RCT: Randomized Controlled Trial; IV: Intravenous; NS: Intranasal

# **Supplementary Figure 1:Preferred Reporting Items for Systematic Reviews and Meta-Analysis (PRISMA) Flow Diagram**

# **Supplementary Figure 2: Publication Bias Analysis Using Funnel Plot**

| *(A) Funnel Plot Analysis of Total bleeding outcomes*   | *(B) Funnel Plot Analysis of Hematoma*   |
| --- | --- |
| *(C) Funnel Plot Analysis of Hematuria*   | *(D) Funnel Plot Analysis of Blood Transfusion*   |
| *(E) Funnel Plot Analysis of Intervention Required to Stop Bleeding*  | *(F) Funnel Plot Analysis of Hyponatremia*   |

# **Supplementary Figure 3: Leave-One-Out Sensitivity Analysis**

Abbreviations: IV: Inverse Variance; CI: Confidence Interval; Tau²(τ²): Between-study variance; Chi²: Cochran’s Q test; df: degrees of freedom; I²: I² statistic

# **Supplementary Figure 4: Subgroup Analyses of Hematoma Outcome**

## *(A) Subgroup Analysis of Hematoma Outcome by Route of Administration*

## *(B) Subgroup Analysis of Hematoma Outcome by Study Design*

Abbreviations: IV: Inverse Variance; CI: Confidence Interval; Tau²(τ²): Between-study variance; Chi²: Cochran’s Q test; df: degrees of freedom; I²: I² statistic

# **Supplementary Figure 5: Sensitivity Analysis of Hematoma Outcomes**

Abbreviations: IV: Inverse Variance; CI: Confidence Interval; Tau²(τ²): Between-study variance; Chi²: Cochran’s Q test; df: degrees of freedom; I²: I² statistic

# **Supplementary Figure 6: Forest Plot Analysis of Safety Outcome: Hyponatremia**

Abbreviations: IV: Inverse Variance; CI: Confidence Interval; Tau²(τ²): Between-study variance; Chi²: Cochran’s Q test; df: degrees of freedom; I²: I² statistic
